# Long non-coding RNA transcribed from pseudogene PPIAP43 is associated with radiation sensitivity of small cell lung cancer cells

**DOI:** 10.3892/ol.2019.11202

**Published:** 2019-12-10

**Authors:** Shilong Wang, Jinming Yu

Oncol Lett 18: 4583-4592, 2019; DOI: 10.3892/ol.2019.10806

Subsequently to the publication of the above paper, the authors have realized that the Abstract of their article contained a few errors that were not picked up upon during the pre-press stages. First, on p. 4583, line 7, the sentence that starts on this line erroneously referred to the radio-sensitive sensitive cell line, H128; this sentence should have read as follows: “The results demonstrated that peptidyl-prolyl cis-trans isomerase A pseudogene 43 (PPIAP43) transcription was increased 2-fold in cells irradiated with 2 Gy gamma radiation compared with unirradiated cells in pre-reported radio-sensitive sensitive cell lines H69, H146, H209 and H187. Secondly, the following sentence (“These cells shared 259 upregulated and 96 downregulated RNA transcripts following radiation.”) should not have been included in the Abstract. Lastly, further changes should have been made to the subsequent sentence regarding the cell lines described and the general sentence construction.

Taken in its entirety, the Abstract should have appeared as follows (the pair of sentences containing changes are highlighted in bold):

**Abstract**. Small cell lung cancer (SCLC) is a highly lethal disease. Although radiation therapy is effective for the majority of patients with SCLC, patient sensitivity to radiation varies. The lack of biomarkers impedes advances in targeting radiation-sensitive patients. In the present study, the changes in transcription patterns of SCLC cell lines were evaluated with or without 2 Gy gamma radiation. **The results demonstrated that peptidyl-prolyl cis-trans isomerase A pseudogene 43 (PPIAP43) transcription was increased 2-fold in cells irradiated with 2 Gy gamma radiation compared with unirradiated cells in pre-reported radio-sensitive sensitive cell lines H69, H146, H209 and H187. PPIAP43 was not upregulated 2-fold following irradiation with 2 Gy gamma radiation compared with unirradiated cells in the pre-reported, less sensitive cell lines H526 and D153.** The RNA transcript of PPIAP43 was aligned with the mRNA of peptidyl-prolyl cis-trans isomerase A (PPIA) at 2 sections (3,732 to 3,917 and 5,327 to 5,657 of the PPIA gene) and the sequences were shown to be 96 and 94% similar, respectively. Therefore, PPIAP43 may act as a sponge for microRNAs which bind with the RNA of PPIA. Therefore, PPIAP43 RNA transcription may serve as a potential biomarker of radio-sensitivity of SCLC.

The authors regret that these errors were not identified prior to the publication of this paper, and apologize to the readers for any confusion or inconvenience caused.

